# Insular operculum disconnection and herniation into the parapharyngeal space due to a fetal Galassi Type III arachnoid cyst: a case report

**DOI:** 10.3389/fnins.2024.1419814

**Published:** 2024-10-01

**Authors:** Ping Li, Qin Zhang, Yuantao Yang, Xinting Ji, Rui Zhao, Shuo Gu

**Affiliations:** ^1^Department of Neurosurgery, Hainan Women and Children's Medical Center, Haikou, China; ^2^Department of Neurosurgery, Children's Hospital of Shanghai, Shanghai, China; ^3^Department of Neurosurgery, The First Affiliated Hospital of Hainan Medical University, Haikou, China

**Keywords:** insular operculum disconnection, arachnoid cyst, fetal brain development, Sylvian fissure, greater wing of sphenoid

## Abstract

Arachnoid cysts (ACs) are frequently encountered as incidental findings in the brain, with most cases being asymptomatic and not requiring intervention. However, severe brain malformations caused by ACs are rare. In this study, we describe the case of an 8-day-old female infant with a left mandibular mass that was diagnosed as an insular operculum, which has become disconnected and herniated into the parapharyngeal space through an incompletely ossified greater wing of the sphenoid, caused by a fetal Galassi Type III AC. The newborn also exhibited left hearing impairment, which did not improve at the 6-month follow-up after the cyst peritoneal shunt. This report highlights that ACs that manifest during the early fetal period may protrude from the cranial cavity through an unossified skull, potentially affecting the development of brain tissues.

## Introduction

Arachnoid cysts (ACs) are congenital, benign collections of intra-arachnoid fluid in the intracranial compartment or the spinal canal. Most ACs are clinically asymptomatic, remain stable in size, and are often diagnosed incidentally. However, a small subset of symptomatic ACs is often associated with mass effects (Al-Holou et al., 2010, 2013; Cincu et al., 2007; Jünger et al., 2022; Fisher et al., 2005; Tsitouridis et al., 2002; Ziahosseini et al., 2013; Kulkarni et al., 2021; Khoulali et al., 2021).

In this study, we describe a case of Galassi Type III AC that developed during the fetal stage, leading to disconnection of the insular operculum and its herniation into the parapharyngeal space through an incompletely ossified sphenoid. We used this case to explore why ACs cause the operculum to detach and herniate into the parapharyngeal space, enhancing our understanding of the development of the Sylvian fissure, skull formation, and the origin of ACs.

## Case presentation

### Patient characteristics

**Figure 1:**
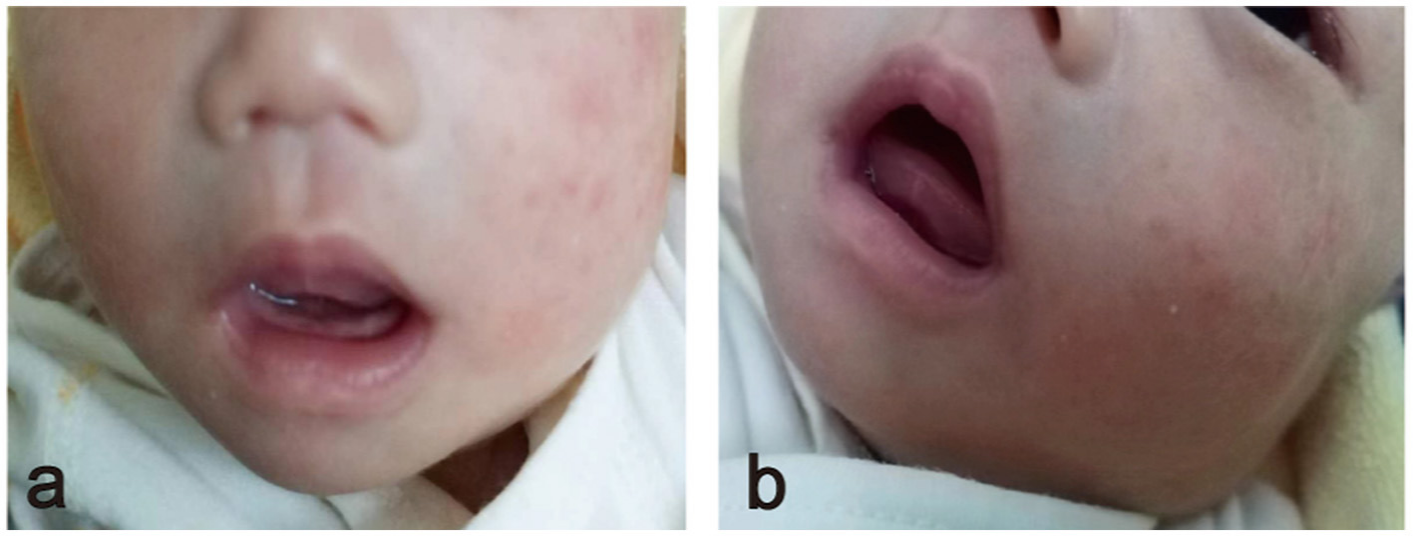
**(a)** Frontal view showing the left lower jaw protruding downward and outward. **(b)** The front lateral view shows the mass located deep in the left jaw.

An 8-day-old female infant presented to our outpatient department with a left mandibular mass that was ~4 × 5 cm in size. The mass was located deep within the left jaw, characterized by a soft texture, poor mobility, and well-defined borders. Notably, the left palpebral fissure was larger than the right, and the eyeball exhibited greater protrusion compared to the contralateral side (Figure 1). During an examination of her oral cavity, it was observed that the left soft palate protruded significantly downward and outward compared to the right side, with a palpable soft mass located above it. The infant showed no signs of dyspnea or feeding difficulties, but her left ear failed the newborn hearing screening. A notable aspect of the case is that both parents were only 14 years old when the child was born, and the pregnancy was unexpected.

Consequently, standardized prenatal checkups were not conducted during the pregnancy. An antenatal examination 3 months before birth showed the presence of an intracranial AC, but the details were unavailable. This child was the firstborn. She was delivered vaginally at a gestational age of 39 weeks and 1 day and a birth weight of 2,750 g. The amniotic fluid was clear at birth, and there was no history of asphyxia rescue. The child's parents had no known history of significant medical conditions, and the mother did not experience any major health issues during the pregnancy. Apart from the intracranial AC and the resulting structural changes, comprehensive systemic and ancillary tests revealed no apparent abnormalities in the cranial structure or other organs. Due to financial constraints and the family's wishes, genetic testing was not performed.

### Clinical findings

**Figure 2:**
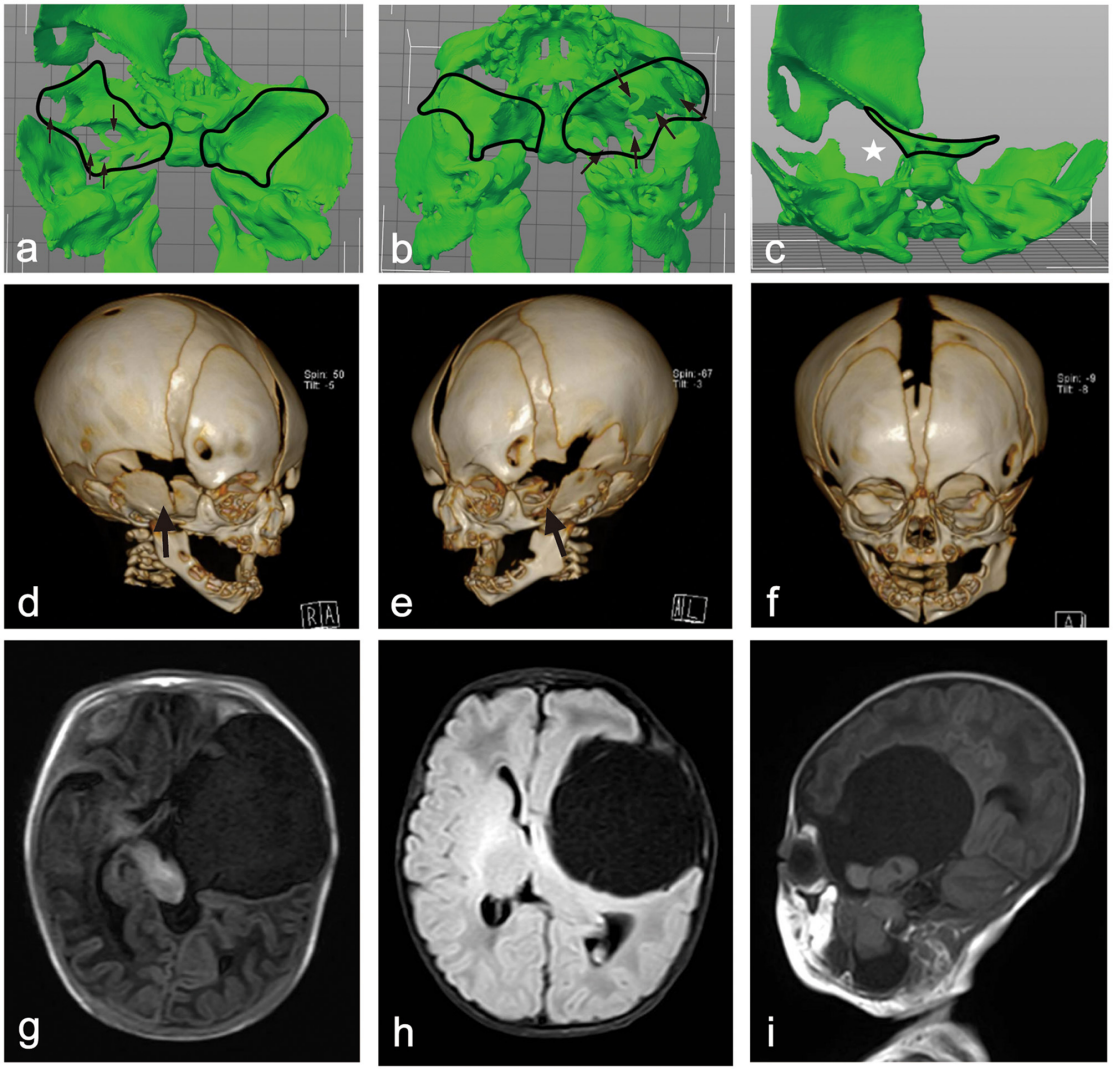
**(a, b)** A 3D model of the skull base above and below view showing multiple defects in the left greater wing of the sphenoid. **(c)** A 3-D model of skull base rear view showing upward of the lesser wing of sphenoid with corresponding left superior orbital fissure was enlarged. **(d, e)** A 3-D CT image of the skull's right and left frontal view shows the right GWS ossified well and a defect on the outside of the left. **(f)** A 3-D CT image of the skull's frontal view shows that the left orbit was enlarged compared to the right side, and the left mandibular head, neck, ramus, and angle were shifted laterally. **(g–i)** Axial T1WI, FLAIR, and sagittal T1WI images show a CSF signal intensity cystic lesion in the left middle cranial fossa and an irregular lesion with brain tissue signal intensity with it. These lesions herniated into the mandibular fossa through the left GWS. The brain tissue and ventricles around the lesion were compressed and displaced, and the midline was also displaced to the right. The left eyeball protruded forward from the contralateral side.

A three-dimensional computed tomography (CT) scan of the skull was performed to assess bone changes associated with the condition. The CT revealed multiple defects in the left greater wing of the sphenoid (GWS) and an upward displacement of the lesser wing of the sphenoid, along with an enlargement of the left superior orbital fissure. The cyst located in the left middle cranial fossa caused irregular compression, outward expansion, and thinning of the surrounding temporal parietal bones. Additionally, the left zygomatic bone was displaced outward and downward due to the pressure exerted by the mass, leading to an enlargement of the left orbit compared to the right orbit. The left mandibular head, neck, ramus, and angle were displaced laterally because of the mass effect on the neck, with the mandibular head dislodged from the fossa and appearing smaller than the contralateral side (Figure 2).

Magnetic resonance imaging (MRI) of the brain revealed a large, well-defined cystic lesion with a thin wall and cerebrospinal fluid (CSF) signal intensity, measuring ~58 × 52 × 93 mm (anterior-posterior × transverse × craniocaudal, respectively), and was located in the left middle cranial fossa, extending into the neck.

This lesion caused significant compression and displacement of the adjacent brain tissue, including the brainstem and lateral ventricle. The midline structure was displaced 5 mm to the right due to the mass effect exerted by the lesion. Within the large cystic lesion, there was an irregular brain tissue lesion measuring ~19 × 22 × 43 mm, which communicated with both the intracranial and neck regions through the greater wing of the sphenoid (GWS) and was completely free from other structures. The left eyeball protruded ~3.5 mm anteriorly compared to the contralateral eyeball. The lesion in the left neck shifted the pharyngeal structure to the right, resulting in compression and displacement of the left submandibular gland. The lower border of this lesion extended down to the level of the inferior aspect of the left mandibular angle (Figure 2).

### Therapeutic intervention

In collaboration with otolaryngologists and maxillofacial surgeons, we determined that middle skull base reconstruction combined with microsurgical resection was the most fundamental therapeutic approach to address this issue. However, due to the complexity of the surgery, the high technical requirements, and the significant risk of postoperative complications, we recommended delaying the radical surgery until an elective date.

Based on our previous experience and a review of the literature, we recognized that children with infantile ACs, with particularly large cysts, are at a higher risk of developing postoperative subdural effusions and/or hydrocephalus following dissection and fenestration. Furthermore, the presence of a brain-like lesion within this patient's cyst further complicated the situation and heightened the risk of postoperative complications. Consequently, we considered a shunting procedure, which posed a lower risk and offered a better safety profile, to be the more prudent surgical approach.

Ultimately, due to financial constraints, the patient underwent a cysto-peritoneal shunting procedure with a constant pressure valve set at 70 mmH_2_O rather than an adjustable shunt valve. Postoperatively, we found the cyst sac fluid to be clear and under high pressure, with normal cell and cerebrospinal fluid (CSF) counts.

### Follow-up and outcomes

The postoperative recovery of the patient was uneventful. The child's postnatal development proceeded similarly to other children of the same age, with no observed developmental delays. At the 6-month follow-up, her left eyeball still protruded ~3 mm more than the contralateral side, although a bilateral fundus examination revealed no significant abnormalities. Hearing loss was confirmed on the left side, but her swallowing and breathing remained unaffected.

**Figure 3:**
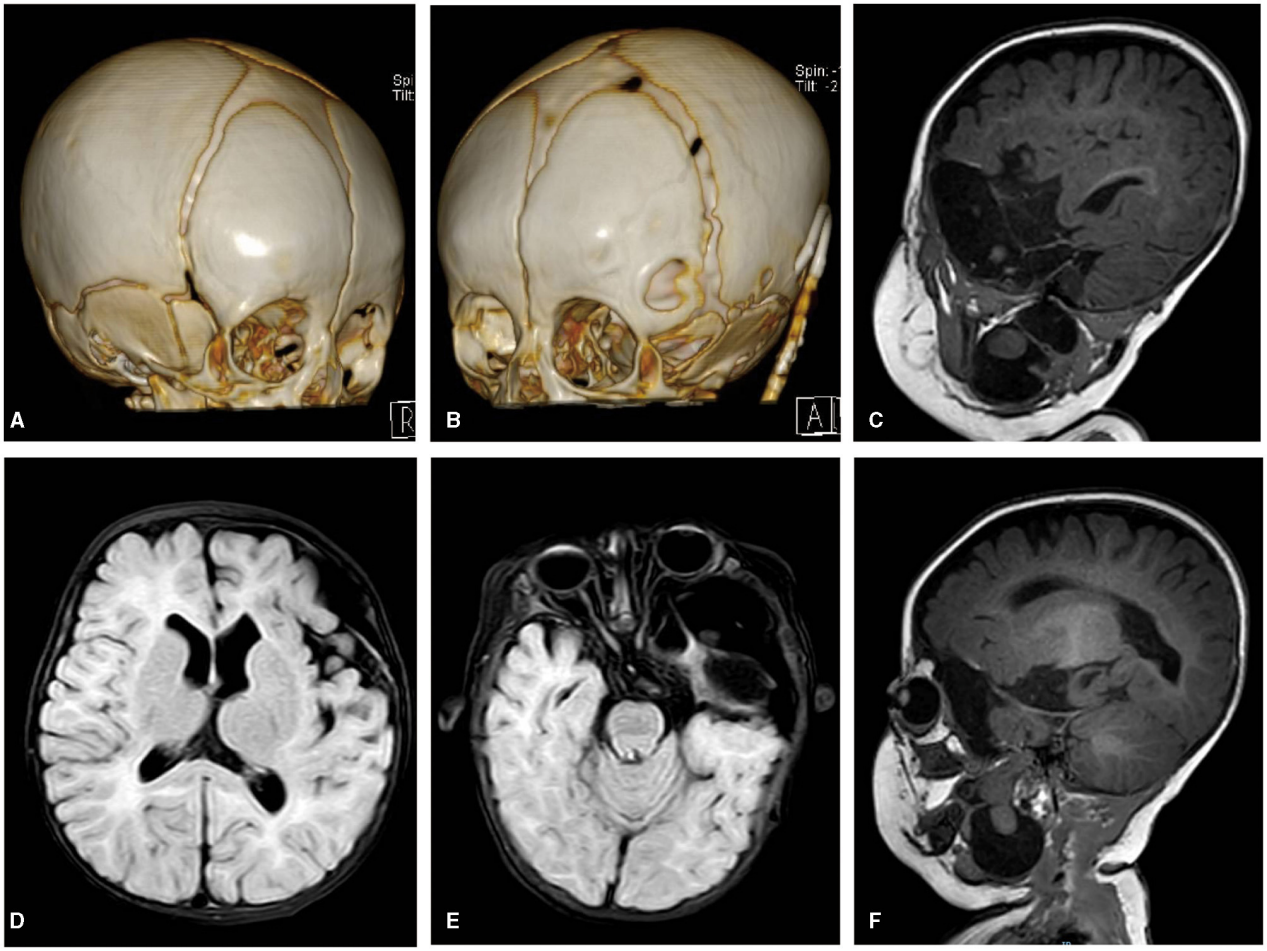
**(A)** A 3-D CT image of the skull's right frontal view showing the right GWS ossified well. **(B)** A 3-D CT image of the skull's left frontal view showing there was a defect on the outside of the left GWS and the tube of the CP shunt. **(C–F)** FLAIR axial and sagittal T1WI images showing a cystic lesion with CSF signal intensity in the left middle cranial fossa were significantly reduced, and correspondingly, the intracranial compression structure was also significantly relieved compared with the preoperative period. There was a partial loss in the left insular opercula and the temporal pole. The brain tissue signal intensity lesion in the cyst still communicated with both parts of the intracranial and neck through GWS.

CT imaging showed that the left GWS remained defective, with partial ossification on its outer side, while the right side was fully ossified (Figure 3). Brain MRI at the 6-month postoperative follow-up revealed a significant reduction in the cyst within the middle cranial fossa, along with decreased intracranial compression compared to the preoperative period. However, the left cerebral cortex remained difficult to identify due to the severe compression caused by the lesion before surgery.

The MRI at 6 months postoperatively revealed a partial loss in the left insular operculum and temporal pole. The brain tissue signal intensity lesion in the cyst appeared to be mildly larger than before, measuring ~9 × 24 × 50 mm, but it continued to connect parts of the intracranial region and the neck through GWS. In addition, we found that the intralesional cyst in the parapharyngeal space had become separated (Figure 3).

## Discussion

ACs are frequently encountered during brain imaging. Most of these lesions are asymptomatic and do not require any surgical intervention. The location of ACs is variable, though they most commonly occur in the middle cranial fossa. Several theories have been proposed to explain the origin of ACs, including agenesis of a part of the brain, minor aberrations in the development of the arachnoid, developmental defect in the condensation of the mesenchyme, and abnormalities in CSF flow (Cincu et al., 2007).

Robertson et al. suggested that cysts in the middle cranial fossa are associated with temporal lobe hypogenesis. Embryologically, the subarachnoid space forms by the expansion of the intercellular space in the meninx primitiva surrounding the neural tube and the clearance of cellular elements in the meninx between fetal ages 6.5 and 8 weeks. The neocortex is present by fetal week 8, with waves of ventriculocortical cellular migration occurring between fetal weeks 7 and 15. It is conceivable that the cyst, caused by the arachnoid membrane, leads to temporal lobe hypogenesis. It is also plausible that the arachnoid coverings of the temporal and frontal lobes do not coalesce during the formation of the Sylvian fissure in early fetal development, leading to the creation of a non-communicating fluid compartment enclosed by arachnoid membranes (Cincu et al., 2007; Robertson et al., 1989).

In our patient, we observed that the enlarged AC within the Sylvian fissure exerted pressure on the adjacent brain tissue, contributing to temporal lobe hypoplasia, which eventually resulted in a portion herniating into the mandible through a defect in the GWS.

The skull base comprises the occipital, sphenoidal, ethmoidal, temporal, and frontal bones. Prior research has indicated that the skull base primarily ossifies through cartilage formation. By week 4 of pregnancy, mesodermal and neural crest cells begin forming the cartilage and bony components of the skull base. By week 8, the skull base consists mostly of the cartilage, with ossification advancing anteriorly from the occipital bone. The initial ossification center emerges at the occipital bone around week 12 of embryonic development, progressing posteriorly to the sphenoidal bone, anteriorly to the frontal bone, and finally to the ethmoidal bone.

The ossification process of the sphenoid bone is particularly complex, involving as many as 19 distinct ossification centers. Embryologically, the body of the sphenoidal bone and a section of the GWS near the foramen rotundum ossify through the cartilage, whereas the rest of the GWS and the pterygoid plates ossify through membranes. Ossification of the sphenoid bone begins around week 13, with the lesser wing forming between weeks 16 and 24 and ossification beginning around week 15.

The foramen ovale and foramen spinosum are key defects in the GWS. These openings in the skull base are formed near existing structures, prompting the surrounding mesenchyme to develop cartilage around the pre-existing blood vessels and cranial nerves (Nemzek et al., 2000; Conley and Phillips, 2017; Chong et al., 2002; Sharma et al., 2017; Jurkiewicz et al., 2007; Leblanc et al., 1991).

A CT scan of our patient revealed multiple defects associated with the GWS, allowing the cyst to herniate from the cranial cavity into the parapharyngeal space, accompanied by a brain-tissue-like lesion. Furthermore, well-ossified bone was observed between these defects. We speculate that the cyst, along with its brain-tissue-like lesion, herniated into the parapharyngeal space prior to the ossification of the GWS, with subsequent gradual ossification occurring around the lesion.

Determining the cause of the intracranial abnormalities in our patient was challenging due to the absence of antenatal findings. After surgery, the intracranial cyst gradually decreased in size, allowing for progressive development of the surrounding brain tissue and relief from compression. This reduction enabled clearer observations of the intracranial brain tissue.

An MRI of the brain revealed that the AC and its contents had herniated into the parapharyngeal space through the GWS, becoming segregated from the surrounding normal brain tissue. Within the cortex, we observed underdevelopment in the left temporal pole, temporal insular operculum, and frontal insula operculum compared to the contralateral side, although the temporal horn of the ventricle was present.

We hypothesized that the development of a Galassi Type III AC during the fetal period influenced the growth of the temporal and frontal lobes, leading to the isolation of the affected brain tissue within the cortex and possibly encasing it within the cyst. The delayed development of the skull base's bone structure may have allowed the cyst and its contents to herniate into the parapharyngeal space, followed by subsequent ossification of the GWS around the cyst.

## Conclusion

ACs frequently occur in benign intracranial anomalies, with most cases being asymptomatic. However, in some instances, ACs can exert mass effects, leading to the compression of surrounding tissues and even remodeling of the skull. The occurrence of an AC accompanied by insular operculum disconnection and herniation into the parapharyngeal space is an uncommon phenomenon. Our case highlights that ACs can manifest during the fetal period, potentially herniating beyond the cranial cavity through an unossified skull, thereby affecting the development of brain tissues.

## Data Availability

The raw data supporting the conclusions of this article will be made available by the authors, without undue reservation.
